# Underweight and overweight in primary school children in eThekwini district in KwaZulu-Natal, South Africa

**DOI:** 10.4102/phcfm.v3i1.203

**Published:** 2011-03-24

**Authors:** Threethambal Puckree, Pooveshni Naidoo, Prabashni Pillay, Therona Naidoo

**Affiliations:** 1 Department of Physiotherapy, University of KwaZulu-Natal, South Africa; 2 Faculty of Health Sciences, Durban University of Technology, South Africa

## Abstract

**Background:**

The prevalence of overweight and obesity in children has been increasing worldwide. South Africa has minimal data on childhood body weight.

**Objectives:**

This study determined whether school children in the eThekwini district in KwaZulu-Natal, South Africa, were underweight or overweight.

**Method:**

A survey with quantitative and qualitative components was conducted amongst 120 participants between 10 years and 12 years of age. The participants were randomly selected from six public schools in an urban district of the province. A calibrated Goldline bathroom scale was used to measure body weight and a KDS Freo non-elastic measuring tape was used to measure height. A questionnaire consisting of open and close-ended questions collected demographic and lifestyle information. Body mass index (BMI) was calculated from height and weight data. Proportions of obese, overweight and underweight children were calculated and subjected to chi-square tests at the *p* ≤ 0.05 significance level. All qualitative information was summarised.

**Results:**

According to World Health Organization criteria, 66% of the children were underweight, 28% were of normal weight and 5% were overweight. The proportion of underweight children increased with age (64% of children aged between 10 years and 11 years vs 70% for 12-year-olds). Of the underweight children, 41% were female and 51% were Indian. Only one child was obese. BMI was related to dietary patterns and activity levels during and outside school hours.

**Conclusion:**

A significant number of primary school children from the six selected public schools in the eThekwini district were underweight. More effort is required to improve the nutritional status of school children in the eThekwini district.

## Introduction

### Setting

#### Key focus

Overweight and obesity amongst school children have reached epidemic proportions in both developed and developing countries, but few published reports have examined its prevalence and significance in South Africa.^1, 2^ The majority of the South African population experiences poor economic status and grapples with the problems of underweight and malnutrition, which is often overshadowed by other epidemics such as obesity.

#### Background and trends

Obesity and overweight amongst children have received attention from policy makers and health ministries, researchers, academics, parents and the World Health Organization (WHO). Also the prevalence of underweight in children has received attention from international health agencies, but seems to be overshadowed by the epidemic status afforded to overweight and obesity. Both over- and underweight are linked to morbidity and mortality in children worldwide, but published literature have focused mainly on children from developed countries.^3^ The statistics on childhood obesity and overweight in Australia, the USA, Portugal, Spain and other affluent or affluence- attaining countries are alarming.^3, 4, 5^ Armstrong, Lambert, Sharwoord and Lambert^6^ found that the prevalence of obesity among South African children was 3.2% for boys and 4.9% for girls, whilst the prevalence of overweight was 2.4% for boys and 10.9% for girls. Goedecke, Jennings and Lambert^2^ reported an overweight prevalence of 17.1% amongst urban South African children between 1 year and 9 years old, while Jinabhai, Taylor and Sullivan^7^ reported the prevalence of overweight to range from 0.4% to 13.3% amongst rural school children between 8 years and 11 years old. Strategies to identify the causes and manage the problem have also received attention from government, the academic community, researchers and the public at large.^1, 2, 3, 4, 5, 6, 7, 8, 9, 10, 11^ Obesity transcends race, culture, socio-economic status and age.^12^ On the other hand, underweight is typically linked to poor countries and children with emotional problems in developed countries.^13, 14^ The emphasis on childhood obesity in the lay and scientific literature distracts one from the problems associated with underweight, the causes of which are different in developed and developing countries.^13, 14^

The attention given to childhood obesity in other parts of the world has also highlighted the situation in South Africa.^1, 2, 3, 6^ It is also known that underweight is related to syndromes of malnutrition, such as kwashiorkor.^7, 15^ Whilst the South African scientific literature on the association between malnutrition and underweight is sparse, it is important to develop a database on the extent of the problem in this country so that alongside overweight prevention, appropriate measures can be intensified to curb the problem of underweight in a country that is considered to be developed compared to the rest of Africa. It is also known that malnutrition and underweight continue to be significant in developing countries,^13^ as evidenced by a study regarding the prevalence of underweight among adolescent girls in a developed country such as Sweden.^14^

Whilst Armstrong et al.^6^ reported on the prevalence of overweight and obesity in South African children, no mention was made of the prevalence of underweight among the studied cohort. Similarly, Jinabhai et al.^7^ reported only on stunting, overweight and obesity in rural South African children.

#### Objectives

The primary purpose of the study was to determine the prevalence of underweight, overweight and obesity among children from public primary schools in an urban district in KwaZulu-Natal. WHO criteria^16^ and those advised by a local paediatrician were used to categorise children based on their body mass index (BMI). The study also allowed for the prevalence of overweight and underweight to be compared and the identification of risk factors, if any.

#### Contribution to the field

By highlighting the existence of underweight alongside overweight amongst school children, the need for provincial and national studies will be revealed. The need for district- wide strategies to manage both underweight and obesity as part of school curricula will be emphasised. In essence, child health will be shown to require more attention.

## Ethical considerations

The Ethics Committee of the Faculty of Health Sciences at the University of KwaZulu-Natal approved the study.

### Potential benefits and hazards

The study posed no hazards to the children. The benefits include extending the existing nutrition programme to more public schools and establishing structured physical education programmes at schools. All data collection sheets were kept confidential.

### Recruitment procedures

Participation was voluntary upon consent of the children's parents or guardians. No incentive was provided and participants were allowed to withdraw if they wanted.

### Informed consent

The procedure was explained to participants using an information sheet. They were then asked to take the consent forms and information sheet home to obtain parental or guardian consent. Each participant retained a copy of the form and information sheet.

### Data storage

Participants were informed that the data collection sheets would be stored at the first author's office at the University of KwaZulu-Natal. Only the authors of this publication had access to the collected information.

## Method

### Materials

A random sample of 50 children between 10 years and 12 years of age (in Grade 6 or 7) from each school was invited to participate in the study, regardless of gender or ethnicity. The randomisation was achieved by allocating a number to every potential participant and selecting 50 of these numbers from a hat.

### Setting

KwaZulu-Natal is one of nine provinces in South Africa. It consists of 11 districts, which are classified as urban, rural, semi-urban or semi-rural. Durban is a city in the urban eThekwini district in KwaZulu-Natal.

### Design

A cross-sectional population-based survey with quantitative and qualitative components was undertaken in the urban eThekwini district of KwaZulu-Natal, South Africa, in 2006. Three suburbs which represented a mix of socio-economic groups were conveniently chosen for sampling. The researchers live in the chosen suburbs. Although Durban has several private and public (state-funded) schools, this study was conducted in public schools. Two schools from each of three selected suburbs were randomly chosen, resulting in six participating schools. All the schools from a suburb were numbered and two numbers were randomly selected.

### Procedure

A total of 300 consent forms were distributed to children in the participating schools. Children were requested to obtain permission from their parents or guardian to participate in the study. Children who did not obtain parental consent, personally refused to participate in the study or did not return their consent forms were excluded from the study. A final cohort of 120 children was eligible to participate in the study. Ethical clearance was obtained from the institutional ethics committee and approval from the Department of Education and principals of the chosen schools was also obtained.

Height (in metres) was measured using a non-extendable, non-elastic, KDS Freo wind-up measuring tape. The investigator ensured that the children stood with their head, shoulders and heels placed firmly against a wall during height measurements. Weight (in kilograms) was measured with a calibrated Goldline bathroom scale. An investigator- administered questionnaire, which consisted of closed and open-ended questions based on existing literature, was used to gather information about the children's eating habits, activity levels and general lifestyle. Reliability of the questionnaire was assured by one investigator administering the questionnaire to all participants. Validity of the questionnaire was guaranteed by conducting a pilot study among five randomly selected children.

### Data analysis

Weight and height measurements were used to calculate BMIs. Children were classified as underweight, overweight or obese using the guidelines from a local paediatrician and the WHO. According to WHO guidelines, a person is regarded as obese if the BMI is equal to 30 or more, overweight if it is between 25 and 29.9, normal if it is between 18.5 and 25, and underweight if it falls below 18.5. The local paediatrician's guidelines were, (1) > 30 for obese, (2) 25–29.9 for overweight, (3) 20–24.9 for normal weight and (4) < 19.9 for underweight. The local norms were important to allow for comparison with world data and to make the findings locally relevant.

Information regarding the children who were obese, overweight or underweight was extracted from the qualitative data. Data were also stratified by race and gender. Percentages were calculated in each of the above categories. Data were analysed using SPSS for Windows (version 16) and chi-square testing with a significance of *p* ≤ 0.05. All qualitative information was also thematically analysed to give further insight into the participants’ lifestyles.

## Results

In the first phase of the study, 187 of the 300 children selected for participation, returned their consent forms. From this group, 120 participants were randomly selected.

The proportion of participants by race and gender, from each of the six selected schools that participated in the study is shown in table 1. Sixty per cent of the participants were female. Twenty-four per cent were Black, whilst the remainder were Indian.

**TABLE 1:** Demographic information of the participants from the six selected schools

School	Participants		Race	
	
	*n*	Female	Black	Indian
1	35	17	4	31
2	13	11	6	7
3	24	15	0	24
4	14	8	0	14
5	21	12	9	12
6	13	9	9	4

**Total**	**120**	**72 (60%)**	**28 (24%)**	**92 (76%)**

*n*, number of participants both male and female

As shown in Table 2, the mean height and weight of the participants increased with age. However, the mean BMI was significantly higher amongst 10-year-old children than amongst either the 11-year-olds or 12-year-olds. The overall mean BMI was well below the WHO standard for normal BMI. It should be noted that the WHO standard is generic and that the Indian standard (as used in India) is specific to people in that country and cannot be used to classify obesity in Indian children in South Africa.^17^

**TABLE 2:** Mean (± s.d.) height, weight and body mass index of participants organised according to age

Race and gender	*n*	Height (m)	Weight (kg)	Body mass index
**10-year-olds**				
Black, female	0	0.00	0.00	0.00
Indian, female	17	1.42 ± 0.11	36.11 ± 11.4	17.94 ± 4.16
Black, male	1	1.31	24	14
Indian, male	7	1.45 ± 0.11	38.29 ± 16.47	17.66 ± 5.54
**Total** (Range)	**25**	**1.43 ± 0.38** (1.21–1.66)	**36.24 ± 9.81** (20–57)	**18.67 ± 4.51** (12.4–26.5)
**11-year-olds**				
Black, female	4	1.46 ± 0.1	40.50 ± 7.85	18.93 ± 3.05
Indian, female	15	1.48 ± 0.05	38.33 ± 9.07	17.38 ± 3.63
Black, male	3	1.40 ± 0.08	32.67 ± 8.32	16.63 ± 2.53
Indian, male	19	1.48 ± 0.06	40.16 ± 11.09	18.43 ± 4.34
**Total** (Range)	**41**	**1.47 ± 0.07** (1.32–1.60)	**38.98 ± 9.81** (31–67)	**17.96 ± 3.81** (13.2–30.2)
**12-year-olds**				
Black, female	14	1.47 ± 0.09	39.07 ± 8.71	17.82 ± 3.26
Indian, female	22	1.51 ± 0.07	41.59 ± 8.44	18.29 ± 3.25
Black, male	6	1.42 ± 0.1	38.67 ± 16.37	18.58 ± 5.07
Indian male	12	1.50 ± 0.06	36.92 ± 7.15	16.60 ± 2.75
**Total** (Range)	**54**	**1.48 ± 0.08** (1.27–1.62)	**39.57 ± 9.22** (30–69)	**15.5 ± 0.71** (13.5–28.1)

**Total (Range)**	**120**	**1.47 ± 0.08 (1.27–1.66)**	**38.68 ± 10.25 (20–69)**	**17.85 ± 3.72 (12.4–30.2)**

*n*, number; s.d., standard deviation.

The proportion of children who were classified as underweight, overweight and having normal weight according to guidelines set by the local paediatrician and the WHO. According to both sets, the majority of the participants (66%) were underweight, of whom 15% were Black and 51% were Indian. Forty-one per cent of the female participants (*p* = 0.03) were underweight. The proportion of underweight children increased significantly with age (*p* = 0.04), with 64% of 10-year-olds and 70% of 12-year-olds being underweight (Table 3).

**TABLE 3:** Percentage of participants in each body mass index category according to guidelines by a local paediatrician and the World health organisation (WHO).

Age (years)	Body mass index guideline	Persentage		

		Underweight	Normal	Overweight
10	Paediatrician	14.2	5.000	1.70
	WHO	12.5	6.000	1.70
11	Paediatrician	24.2	9.200	0.83
	WHO	22.0	12.00	0.83
12	Paediatrician	35.8	5.700	2.50
	WHO	31.7	10.83	2.50

**Total**	**Paediatrician**	**74.2**	**19.90**	**5.03**
	**WHO**	**66.2**	**28.83**	**5.03**

Note: Data exclude that of one child who was obese.

According to WHO guidelines (which are generic and may not provide accurate cut-off values for South African children) 5% of the children were overweight. Similar results were obtained using either the local paediatrician's or the WHO guidelines.

### Qualitative responses

#### Diet

Eighty-six per cent of the children reported that they consumed nutritious meals, such as cereals, yoghurt and fruit. One child reported to have pies and sometimes burgers for lunch. Some children (6.3%) did not eat nutritious meals whilst 7.6% of the children did not consume anything at school. More than half of the participants (54.6%) indicated that they frequently purchased items from the school tuck shop more than three times a week. Of these children, 83.2% bought items that could be considered ‘junk food’ or not nutritious. More than half the respondents (55.5%) stated that they seldom had snacks between meals, whilst 37% often had snacks. When questioned on how regularly takeaway food was consumed, 90% stated once or twice a week, whilst 84% responded to have it more than twice a week.

A relationship between weight deviation and dietary habits was noted, with both overweight and underweight children reporting non-nutritious eating habits. Of the seven children found to be overweight, one was obese. More than three-quarters (76.7%) of these children obtained items that were not nutritious. It was also noted that 90.6% of the overweight children regularly had snacks between meals. In addition, almost all (97.6%) of these children often ate takeaway foods.

#### Physical activity

Almost three-quarters (72%) of the children were driven to school. One school did not include physical education in their curriculum. All other schools included one 30-minute physical education session per week in their syllabus. Almost all the children (92%) participated in extracurricular sporting activities, yet there was a large percentage (74%) of children who found themselves often fatigued. All children watched more than 4 hours’ television on weekdays and 6 – 8 hours on weekends. When asked about their opinions regarding the quality of their lifestyles, 69% of participants considered themselves healthy.

Four of the children found to be overweight, did not walk to school. One of these children did not participate in extracurricular sporting activities. Four of the seven overweight or obese children regularly complained of fatigue.

## Discussion

### Outline of results

This study showed that a significant proportion of 10 – 12-year-old primary school children from the selected schools in eThekwini, KwaZulu-Natal, were underweight. Only one of the 120 children was obese and only 5% were overweight according to WHO standards. These results differ from those of an earlier South African study^6^ in which 3.2% of boys and 4.9% of girls were obese, and 2.4% of boys and 10.9% of girls were overweight. However, Indian children were not included in the data analysis in that study, whereas 76% of the participants in the present study were Indian. In a study among children in the North West Province, South Africa, Goedecke, Jennings and Lambert^2^ found that 17% of the respondents were overweight. Research in Australia showed that by 1997, approximately 16% of boys and 18% of girls were overweight.^10^ In the USA, a study between 1999 and 2000 showed that 17% of boys and 14% of girls between 6 years and 11 years old were overweight.^4^

The difference by gender and race was not significant (*p* = 0.1) amongst the 66% of children who were found to be underweight in our study. However, BMI decreased with age. The findings show that pockets of South African children may be experiencing a nutritional problem similar to that in developing countries.^13^ Despite South Africa being considered to be a developing country, one finds first and third world socio-economic conditions throughout the country.

Effects of cultural differences on dietary patterns may also need to be explored. The cohort consisted of Black and Indian South Africans, without gender or race differences amongst the large proportion of underweight children. It is not known whether daily nutritional requirements as per the food pyramid were met, which could have contributed to the observed proportion of underweight participants. Normal daily nutritional requirements may be beyond the financial means of many of the participants. As stated previously, state-funded nutritional programmes that are in place for schools in disadvantaged areas may need to be explored. The increased emphasis on childhood and adult obesity as reported in an earlier US National Health and Nutrition Examination Survey^4^ suggest that overweight and obesity continue to worsen as age increases. Overweight children are at risk of becoming overweight adults and are more likely to experience a higher morbidity associated with excess weight.^4^ Similar to the morbidity associated with overweight and obesity,^6^ underweight could impact developing children, preventing them from reaching their full potential.

The large percentage of girls found to be underweight (41%) could be due to biological, social, emotional, behavioural or developmental factors.^18^ It was also noted that 66% of the children were underweight. This was probably due to adolescence, peer pressure, poor eating habits or emotional factors as explained by Hossain, Kawar and El Nahas^13^ and Werner, Magnuson and Bodin.^14^

The decrease in BMI with increasing age may require investigation. Amongst Swedish children^14^ peer pressure and the need to maintain an acceptable physical appearance were factors which resulted in underweight. In a developing country where poverty is recognised and the government is considering a basic income grant for the poor, underweight among children should be researched in more depth and addressed appropriately. In 1978, Epstein, Masek and Marshal^19^ implemented a colour-coded, energy-based programme, which improved students’ knowledge of the energy value of specific foods. Our questionnaire showed that participants did not follow a specific dietary plan, but rather made regular purchases from the tuck shop and often consumed takeaway foods and snacks. This may also hold true for the small percentage of participants who were found to be overweight, as mentioned earlier. The fact that participants felt the need to buy snacks from the tuck shop could be the result of a range of factors, which were not investigated. Although it was noted that most individuals had nutritious meals for breakfast and lunch, there is room for further investigation. Good nutrition in childhood is essential to ensure long-lasting quality of life. The shift to westernised lifestyles during recent years has resulted in poor dietary habits amongst both children and adults.^20^

Unhealthy eating habits are a modifiable cause of overweight or obesity.^3^ Frequent purchases of non-nutritious meals by children may be attributed to their over-exposure to unhealthy food in the media (e.g. advertisements for highenergy foods like hamburgers, cold drinks and chocolates). Owing to the over-consumption of such foods and eating patterns when bored or doing homework, a relationship between dietary habits and children’s health status can be found.^21^

Fitness is another important factor related to health outcomes, independent of physical activity.^22^ Our results showed that 72% of the respondents were transported to school and, notably, also the overweight respondents. Even though 82% of the children had physical education included in their curriculum, the duration of sessions was short (only 30 minutes once a week). US-based research has shown that many children are exposed to physical education only once or twice a week, or not at all.^9^ Our data showed that the duration of physical education sessions ranged from 15 to 30 minutes, indicating similar trends for the duration of physical education as in the USA. A lack of physical activity is considered to be a leading cause of overweight and obesity.^23^ Although 92.4% of the children participated in extracurricular sporting activities and just 11.6% of the overweight subjects were inactive, it was difficult to grade their activity levels owing to the type of sport played as well as the duration. Recent data have shown that many diet and exercise interventions were found to be ineffective in preventing weight gain.^24^

Close to three-quarters (74%) of the participants reported being fatigued regularly. Whilst the majority of these were underweight, 65% of the overweight participants were also found to be easily fatigued. Recent studies related to medical conditions have shown that excess weight on the chest wall region decreases the lung volume and chest wall compliance. An increased weight on the chest wall (known as chest wall loading) requires increased effort of breathing owing to altered pulmonary function.^25^

All participants indicated that they often watched television or played computer games for more than 4 hours a day. A recent *Healthy People 2010* report identified television viewing as an important contributing factor to weight gain in children who watched 2 or more hours’ television a day. It was found that television viewing is positively related to overweight and obesity. Although watching television is not entirely a surrogate measure for physical activity, it does provide a good estimate for the amount of time that children spend being inactive and correlates very well with energy intake.^4^ Most researchers suggest that for an effective weight loss programme, children need to reduce their energy intake and increase their level of physical activity, as these go hand in hand.^8, 21, 24, 26^

Effective management of overweight or obesity should consist of preventive measures, education, therapy and a follow-up programme.^23^ Preventive measures may range from universal and secondary prevention to tertiary management strategies for overweight or obese children, as described in literature.^8^ Formal exercise programmes, behaviour modification, consultation with a dietician or nutritionist and drug therapy^26, 27, 28^ are all considered means of therapeutic intervention.

### Limitations

The results of the current study suggest caution in generalising conclusions since only a small group of children were enlisted to participate in the study. However, the findings may be attributed to a range of unproven causes. Firstly, the study was conducted in a city in an urban district of KwaZulu-Natal, with a large proportion of a previously rural population now living in informal settlements. The proportion of participants who live in informal settlements is not known. The children were selected from public schools, where school attendance is free or a minimal school fee is leveraged. This type of school attracts children from lower and lower middle income homes. In the South African setting, this usually implies that both parents have to work to sustain the family. However, with the economic downturn, the effects of unemployment could have affected these children. Although these schools can apply for the opportunity to provide a state-sponsored nutritious lunch to eligible children, none of the schools in this sample are known to have subscribed to the scheme.

### Recommendations

It is recommended that a larger number of schools and more children should be included in future studies of this kind. Considering that urban areas were used in this study, it is recommended that overweight and obesity should be investigated across a wider range of different types of school (public and private; urban and rural) to obtain a representative cross-section of South African children. More information on dietary habits and duration and type of sporting activities should be sought. Teams of health care professionals should develop and implement preventive and management programmes to curb the potential economic drain that could result from obesity or underweight. South Africans must develop their own BMI norms and use the WHO criteria for comparison.

## Conclusion

The results showed that a large number of primary school children in the selected schools in eThekwini were underweight. The majority of the participants were Indian and female. It was also evident that as age increased, BMI decreased. No gender or race differences were observed. The data suggest a need to develop specific BMI norms for the South African population similar to that reported for Australian children.^10^

The results of this study highlight underweight amongst children in South African schools. The emphasis on obesity should not override the need to investigate and develop interventions for underweight among South African children.
